# Porous cementless cups combined with trabecular metal augments in revision hip arthroplasty: mid-term outcomes

**DOI:** 10.1038/s41598-025-21990-8

**Published:** 2025-10-31

**Authors:** Egor Polevoi, Sergey Kagramanov, Hamlet Chragyan, Hovakim Aleksanyan, Carlos Barrios Pitarque, Rafael Llombart Ais

**Affiliations:** 1 School of Doctorate, Valencia Catholic University Saint Vincent Martyr, Valencia, Spain; 2 N.N. Priorov National Medical Research Center of Traumatology and Orthopaedics, Moscow, Russian Federation; 3 Institute for Research On Musculoskeletal Disorders, School of Medicine and Health Sciences, Valencia Catholic University Saint Vincent Martyr, Valencia, Spain; 4 Traumacenter Valencia, Hospital “La Casa de Salud”, Valencia, Spain

**Keywords:** Revision total hip arthroplasty, Trabecular augment, Porous cup, Bone defect, Paprosky classification, Trauma, Outcomes research, Skeleton

## Abstract

Reconstruction of acetabular bone defects represents one of the most technically demanding aspects of revision total hip arthroplasty. Although numerous implant options and surgical techniques have been proposed, porous acetabular components combined with trabecular metal augments have gained widespread acceptance as a contemporary reconstructive strategy. The present study aims to assess implant survivorship and to report mid-term clinical and radiographic outcomes associated with this technique. We identified 69 revision total hip arthroplasties (69 patients) performed at the Department of Joint Arthroplasty of the Priorov National Medical Research Center of Traumatology and Orthopedics between January 2016 and June 2022, using porous acetabular cups in combination with trabecular metal augments. Among these patients, 46 (66.6%) were female and 23 (33.4%) were male. The mean patient age was 59.9 years (± 12.4), with a mean follow-up duration of 4.9 years (± 1.78), ranging from a minimum of 2.6 years to a maximum of 9 years. Functional outcomes were assessed using the Harris Hip Score (HHS), Visual Analogue Scale (VAS), and Western Ontario and McMaster Universities Osteoarthritis Index (WOMAC), while implant survival was evaluated using Kaplan–Meier analysis. The overall HHS significantly improved from a preoperative mean of 32.9 (± 9.8) to a postoperative mean of 86.7 (± 4.9). VAS scores decreased from 8.1 (± 0.5) to 3.4 (± 0.5), WOMAC scores decreased from 61.3 (± 4.7) to 24.7 (± 2.4), indicating favorable clinical and functional outcomes (*p* < 0.00001 for each scale). Kaplan–Meier survival analysis demonstrated a 9 years (109.7 months) cumulative implant survivorship rate of 98.55%. The results indicate that porous acetabular cups combined with trabecular metal augments represent an effective option in revision hip arthroplasty, demonstrating excellent mid-term outcomes in terms of both implant survivorship and functional improvement.

## Introduction

The continuously increasing global demand for primary total hip arthroplasty (THA) has been inevitably associated with a proportional rise in revision procedures. According to multiple national joint registries, revision surgeries account for up to 10% of all arthroplasties performed annually, and this proportion continues to grow year by year^1–4^. Among the various indications for revision, aseptic loosening of the acetabular component remains the most common cause, frequently accompanied by extensive periprosthetic bone loss and acetabular bone defects^5–10^.

Reconstruction of the acetabulum during revision THA represents a technically demanding challenge for orthopedic surgeons^11–14^. The selection of an appropriate reconstruction strategy is largely dependent on the severity of the bone defect; greater bone loss typically necessitates more complex reconstruction techniques^15–17^.

Compared to primary THA, revision procedures are associated with significantly higher rates of mechanical failure and the need for subsequent re-revision surgeries. Moreover, each additional surgical intervention often contributes to progressive bone loss, further complicating acetabular reconstruction. Data from the UK National Joint Registry demonstrate a cumulative revision risk of 8.17% at 19 years following primary THA, compared to 21.28% after a revision procedure^2^. Similarly, the Swedish Hip Arthroplasty Register reports a progressive increase in the risk of re-revision in patients who have undergone one or more previous revision surgeries, ranging from 22 to 37%^3^. According to the New Zealand Joint Registry, the revision risk at 18 years is 12.5% after primary THA and increases to 27.5% following revision arthroplasty^18^.

These findings underscore the importance of employing reliable and durable surgical techniques in revision hip arthroplasty. Furthermore, implant systems used in this setting must provide both biomechanical stability and long-term fixation.

Several reconstructive options and implant designs have been proposed for the management of acetabular bone defects. However, the optimal strategy for bone defect management and restoration of the anatomical hip center of rotation remains a subject of ongoing debate in contemporary orthopedic practice.

The introduction of porous metal augments has expanded the possibilities for acetabular reconstruction, especially in cases of critical bone loss. These modular components allow for stable fixation of hemispherical acetabular shells without the need for structural allografts. Augments are available in various geometries and are primarily intended to compensate for segmental or cavitary bone defects. Their modular application in combination with porous acetabular shells has demonstrated promising results, even in the reconstruction of severe bone defects classified as Paprosky types IIIA and IIIB^19–21^.

We hypothesize that the use of porous acetabular components in conjunction with trabecular metal augments may provide a reliable and reproducible solution for complex acetabular reconstructions. The objective of the present study is to evaluate the mid-term clinical and radiological outcomes of such reconstructions, including implant survivorship and postoperative complications.

## Materials and methods

This study (ACTRN12625000667415) is a retrospective analysis of prospectively collected data, conducted using the surgical registry of the Department of Joint Arthroplasty at the Priorov National Medical Research Center of Traumatology and Orthopedics. In our view, every patient scheduled for revision total hip arthroplasty should undergo thorough evaluation to rule out periprosthetic joint infection. Accordingly, all patients underwent a standardized preoperative evaluation to exclude periprosthetic joint infection prior to revision surgery. The diagnostic protocol incorporated clinical, radiographic, and laboratory assessments. Clinical examination included inspection of the skin and soft tissues, with particular attention to the presence of sinus tracts, which were considered an absolute contraindication to a one-stage revision and an indication for a staged procedure. Radiographic assessment included plain radiographs and CT imaging, with a specific focus on the presence or absence of periosteal reactions suggestive of infection. Laboratory markers (erythrocyte sedimentation rate, leukocyte count, C-reactive protein, and procalcitonin) were analyzed in conjunction with intraoperative findings and rapid cytological analysis of synovial fluid (quantitative leukocyte count). If intraoperative findings raised suspicion of infection, surgeons proceeded directly with prosthesis removal and implantation of an antibiotic-loaded cement spacer, thereby converting the procedure into the first stage of a two-stage revision.

**Fig. 1:**
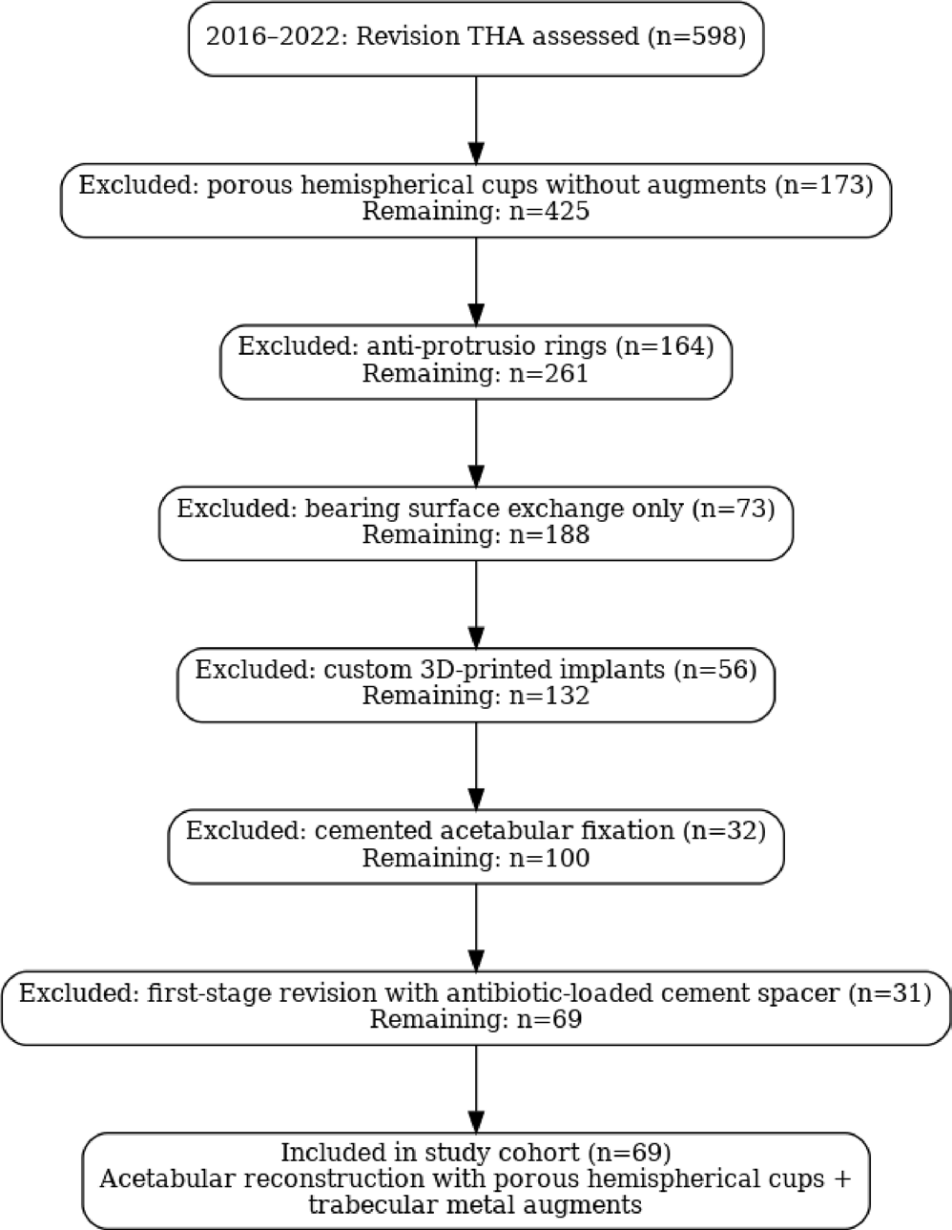
Flow diagram of revision THA cases performed between 2016 and 2022.

The study included all consecutive cases of one-stage revision THA using porous cementless acetabular components in combination with trabecular metal augments performed between January 2016 and June 2022. During this period, a total of 598 revision procedures were perfomed (a variety of surgical techniques and implant types were employed for these procedures), among which 69 patients (69 revision THAs) who underwent a one-stage revision using porous cementless acetabular components combined with trabecular metal augments, all of whom showed no clinical or laboratory evidence of infection, were identified (Fig. 1).

Clinical data, demographic information, and intraoperative findings were extracted from the institutional registry and patients’ medical records for further evaluation. According to departmental protocol, all patients were scheduled for routine postoperative follow-up at 3 months, 1 year, 2 years, 5 years. Additional follow-up assessments were conducted up to 9 years poseoperatively with the most recent data collected in 2025. In cases where a patient was unable to attend the follow-up visit, remote assessment was conducted via telephone interview to evaluate clinical outcomes using standardized departmental questionnaires and protocols. Additionally, patients were requested to submit control radiographs via email for further assessment. All complications requiring re-revision surgery, radiographic signs of implant stability or loosening, as well as the occurrence of reoperations, were documented in the institutional registry.

A total of 69 patients were included in the final analysis. The mean age at the time of revision surgery was 59.9 years (± 12.4), with 46 female and 23 male patients. The maximum follow-up duration was 9 years, with a minimum of 2.6 years and a mean follow-up of 4.9 years (± 1.78). The most common indication for revision surgery was aseptic loosening of the acetabular component with radiographic evidence of migration (55 cases), followed by polyethylene liner wear with associated periacetabular osteolysis (9 cases), and recurrent dislocation of the prosthesis (5 cases). The parameters evaluated in this study included implant types, acetabular bone defect classification according to Paprosky, implant survivorship, and clinical outcomes.

### Preoperative radiographic evaluation

All imaging studies—including anteroposterior pelvic radiographs, focused radiographs of the hip, and multislice computed tomography (MSCT) of the pelvis—were reviewed by orthopedic surgeons at the department. Radiographic evaluation included assessment of bone loss, acetabular bone defects, and the presence or absence of pelvic discontinuity. Particular attention was given to the integrity of the Kohler’s line, the presence of the teardrop figure, the degree of acetabular component migration, the vertical position of the hip center in relation to the inter-teardrop line, and the extent of osteolysis.

Acetabular defects were classified according to the W. Paprosky classification system. Patients were categorized into the following groups (Table 1): Type IIB defects were identified in 22 cases (32%), Type IIIA in 37 cases (54%), and Type IIIB in 10 cases (14%).

**Table 1:** Distribution by groups.

	IIB	IIIA	IIIB
The number of patients	22 (32%)	37 (54%)	10 (14%)
Age	60 (± 13.7)	60.4 (± 10.3)	58.8 (± 17.1)
Male	9	11	3
Female	13	26	7
Time of follow-up (years)	4.9 (± 1.8)	4.8 (± 1.7)	5.6 (± 1.8)

### Surgical technique

**Fig. 2:**
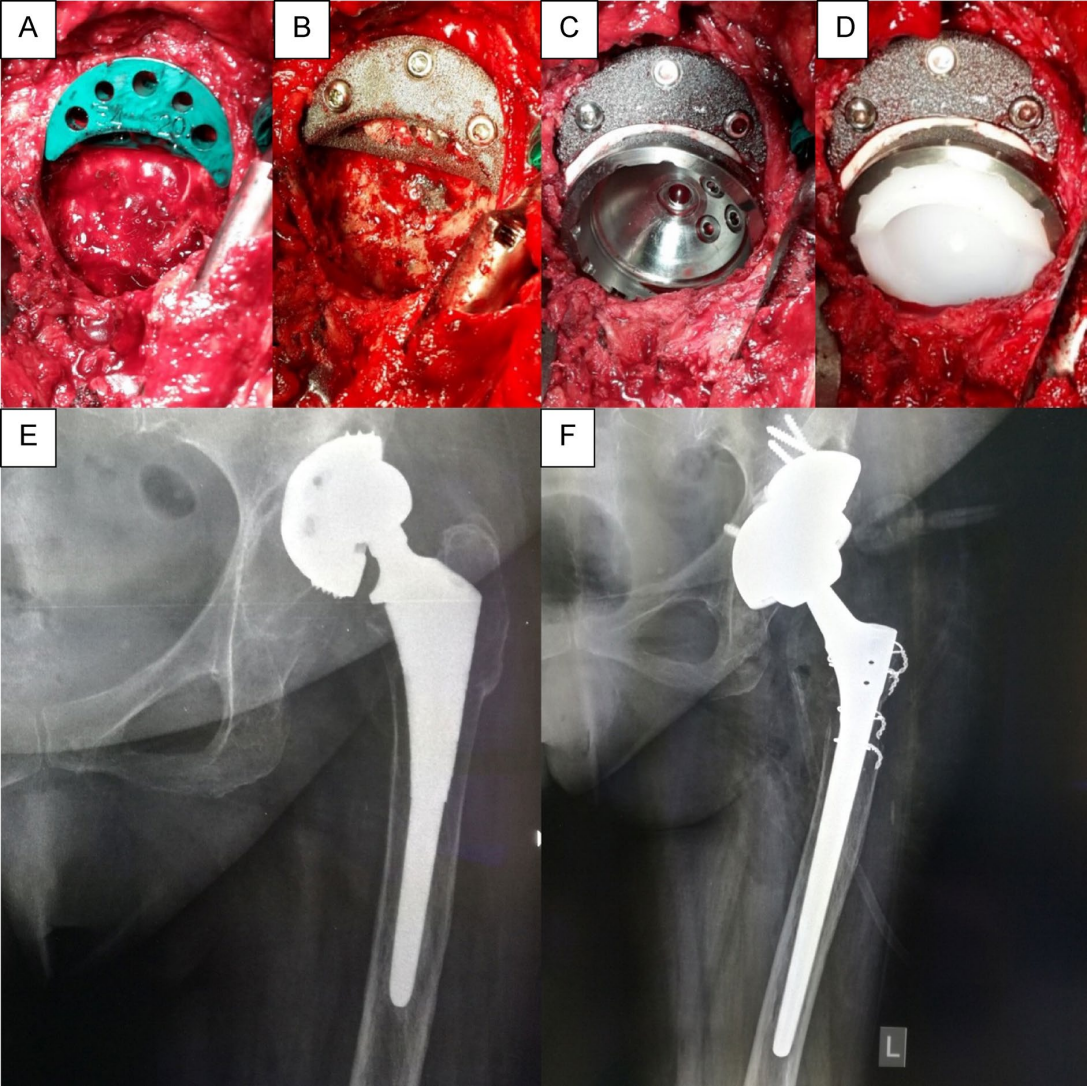
Key steps of the surgical technique in a patient with aseptic loosening and a type IIIA acetabular defect. (**A**) trial fitting of the trial augment, (**B**) fixation of the trabecular augment using three screws, (**C**) implantation of the porous acetabular shell, (**D**) insertion of the liner, (**E**) preoperative X-rays, (**F**) postoperative X-rays.

All procedures were performed by three senior orthopedic surgeons, each with an average annual caseload of up to 300 total hip arthroplasties. A standard anterolateral approach was used in all revision surgeries. Key steps of the surgical procedure are illustrated in Fig. 2. The general surgical protocol was as follows:

1. *Surgical exposure* of the joint with excision of fibrotic scar tissue and meticulous hemostasis. Visual inspection for signs of joint infection was performed in each case; in cases of suspected infection, synovial fluid samples were sent for immediate microscopic analysis, including leukocyte and neutrophil counts. Subsequent surgical steps were undertaken only after the absence of joint infection had been confidently ruled out.
2. *Component removal* included obligatory explantation of the bearing surface components, removal of the femoral stem if indicated, and extraction of the acetabular component. After component removal, the acetabular bone defect was assessed according to the Paprosky classification system.
3. *Trial component placement* was carried out to determine optimal size and orientation of the acetabular augment and final acetabular shell. Reaming was performed with hemispherical reamers to prepare the seating areas for both the augment and the acetabular component.
4. *Implantation of trabecular metal augments* was achieved using screws in a configuration that allowed for subsequent press-fit placement of the acetabular shell. A thin layer of bone cement was applied to the surface of the augment that would contact the cup to enhance stability. The acetabular component was then inserted and additionally secured with supplementary screws.
5. *Insertion of new bearing surface components*, including the polyethylene liner and femoral head, followed by trial reduction of the joint to assess range of motion and implant stability.
6. *Layered wound closure* was performed, followed by application of an aseptic dressing and postoperative compression garments.

The acetabular components that were removed and replaced with revision implants were predominantly uncemented (43 cases), including 22 standard hemispherical cups from various manufacturers, 9 threaded-cups of the Zweymüller type (Bicon, Smith & Nephew, Memphis, Tennessee), 8 threaded-cups ESI (Endoservice, Russia), and 4 CLS Spotorno expansion anchorage cups (Zimmer Biomet, Warsaw, Indiana). In 21 cases, revision surgery was performed for loosening of cemented all-poly cups. In addition, 4 Müller-type reinforcement rings and 1 Burch–Schneider ring were revised.

The porous acetabular components used in revision surgery included the Continuum system (Zimmer Biomet, Warsaw, Indiana) in 50 cases, the Redapt system (Smith & Nephew, Memphis, Tennessee) in 18 cases, and the Pinnacle system (DePuy Orthopaedics, Warsaw, Indiana) in 1 case. Depending on the extent and geometry of the acetabular defect, either one or two trabecular metal augments were used in each case. Augments included TMARS (Zimmer Biomet, Warsaw, Indiana) or Redapt (Smith & Nephew, Memphis, Tennessee), configured either as dome augments (60 cases) or medial footing augments (9 cases), based on individual defect morphology.

Femoral component revision was performed in 39 cases using two types of implants. The first type comprised Zweymüller-style femoral stems, including 6 standard stems (Alloclassic, Zimmer Biomet, Warsaw, Indiana) and 9 revision versions (Alloclassic SLL, Zimmer Biomet, Warsaw, Indiana; SLR, Smith & Nephew, Memphis, Tennessee; Dialoc RS, Implantcast GmbH, Germany). The second type consisted of revision stems with diaphyseal fixation, including 6 Redapt stems (Smith & Nephew, Memphis, Tennessee) and 18 Wagner SL stems (Zimmer Biomet, Warsaw, Indiana). Extended trochanteric osteotomy was required in 3 cases, necessitating additional cerclage fixation at the osteotomy site and partial weight-bearing restrictions for the first 2 postoperative months. This rehabilitation protocol did not differ from that applied to the remainder of the cohort, as all patients undergoing revision with the use of augments were advised to follow limited weight-bearing for up to 2 months postoperatively.

### Postoperative management protocol

In the postoperative period, all patients without exception received subcutaneous anticoagulant injections at prophylactic doses for 30 days, together with compression stockings for the same duration. Prophylactic antibiotic therapy was limited to a single intravenous dose of cefazolin administered within 60 min prior to skin incision; no postoperative antibiotics were administered. Adequate analgesia was provided using non-steroidal anti-inflammatory drugs (NSAIDs), in combination with proton pump inhibitors (Omeprazole) for gastric protection.

Early mobilization was encouraged, with all patients mobilized on the first postoperative day, initially ambulating within the ward using a walker. An orthopedic rehabilitation protocol was prescribed, with partial weight-bearing recommended for up to 2 months, followed by gradual progression to full weight-bearing thereafter.

### Postoperative radiographic assessment

Postoperative radiographic evaluation included analysis of position of the center of rotation, measurement of the inclination angle, and assessment of the presence, absence, or progression of radiolucent lines around the implant.

The position of the center of rotation was measured relative to the teardrop figure. Radiographs were obtained at true scale, which allowed for accurate calculations. An inter-teardrop line was drawn by connecting the teardrops on both sides, and the distance from this line to the center of the prosthetic femoral head was recorded. With respect to the inclination angle of the acetabular component, the inter-teardrop line was again used as a reference, and the angle of cup orientation was determined relative to this line. The appearance or progression of new radiolucent lines in the DeLee–Charnley zones, as compared with immediate postoperative radiographs, was considered a potential sign of implant loosening. Definitive signs of loosening included circumferential radiolucency along the bone–implant interface and changes in the position of the acetabular component.

### Functional outcomes

Functional outcomes were assessed using the Visual Analog Scale (VAS), the Harris Hip Score (HHS), and the Western Ontario and McMaster Universities Osteoarthritis Index (WOMAC) at 1-year, 2-year, 5-year follow-up. These values were compared to preoperative baseline scores.

### Statistical analysis

Data collection and statistical analysis were performed using IBM SPSS Statistics version 24.0 (IBM Corp., Armonk, NY, USA). Qualitative variables were reported as absolute and relative frequencies. Quantitative variables were expressed as means and standard deviations (SD). Comparisons between preoperative and postoperative outcomes (VAS, HHS, and WOMAC scores) were carried out using a paired-samples t-test. Implant survivorship was analyzed using time-to-event methods, where any re-revision surgery, removal of the augment and/or acetabular component, regardless of cause, was considered the endpoint. Kaplan–Meier survival analysis was used to estimate implant survival rates.

## Results

No patients were lost to follow-up; however, 62 of the 69 patients were available for in-person clinical and radiographic evaluation. Seven patients were unable to attend physical follow-up visits and were assessed via telephone interviews, functional questionnaires, and submission of up-to-date radiographs through electronic correspondence.

### Functional outcomes

The overall HHS significantly improved from a preoperative mean of 32.9 (± 9.8) to a postoperative mean of 86.7 (± 4.9). VAS scores decreased from 8.1 (± 0.5) to 3.4 (± 0.5), WOMAC scores decreased from 61.3 (± 4.7) to 24.7 (± 2.4), indicating favorable clinical and functional outcomes (*p* < 0.00001 for each scale).

In patients with Paprosky type IIB defects, the HHS increased from 45.3 (± 5.3) to 89.7 (± 2.8), VAS scores decreased from 7.8 (± 0.5) to 3.5 (± 0.4), and WOMAC scores decreased from 55.8 (± 2.2) to 22.2 (± 1.4).

In patients with type IIIA defects, the HHS improved from 28.7 (± 3.9) to 86.9 (± 3.7), VAS scores declined from 8.2 (± 0.5) to 3.4 (± 0.6), and WOMAC scores decreased from 62.6 (± 1.9) to 26.0 (± 2.0).

For patients with type IIIB defects, the HHS increased from 21.5 (± 2.3) to 79.2 (± 5.6), VAS scores decreased from 8.1 (± 0.5) to 3.25 (± 0.8), and WOMAC scores decreased from 68.5 (± 1.6) to 25.3 (± 1.2).

**Fig. 3:**
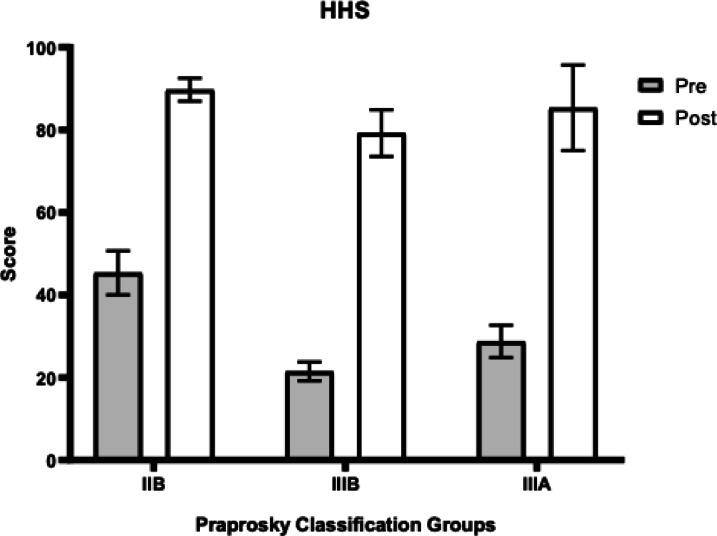
Dynamics of improvement in HHS scores.

**Fig. 4:**
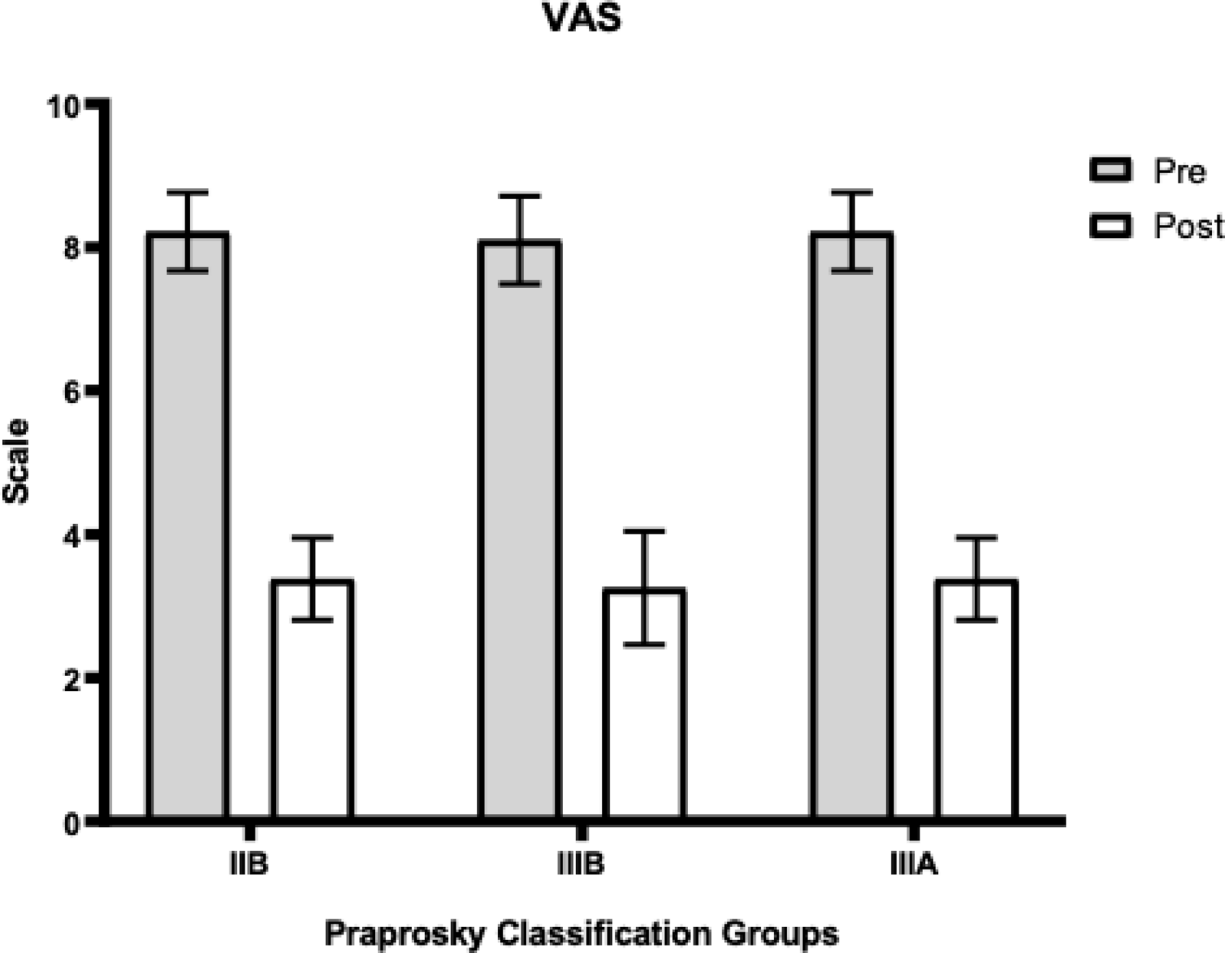
Dynamics of pain reduction according to VAS.

**Fig. 5:**
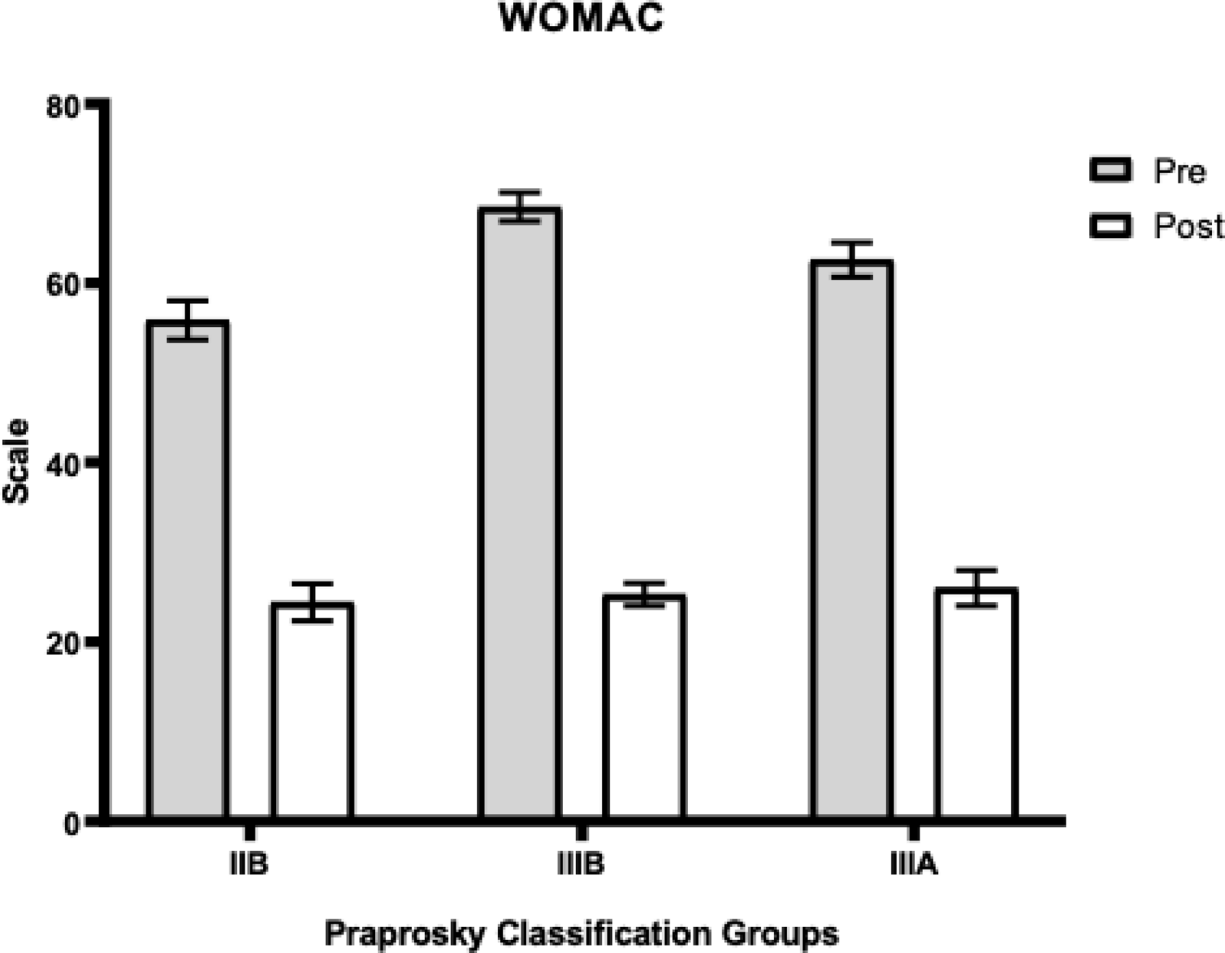
Changes in WOMAC scores.

A statistically significant difference in functional outcomes assessed by the HHS score was observed depending on the severity of the acetabular defect, with the most favorable results achieved in the IIB defect group. Additionally, a statistically significant difference in WOMAC scores was noted between the IIB and IIIA groups. These data are summarized in Table 2. The graphical representation of changes in each scale (HHS, VAS, WOMAC) is shown in Figs. 3, 4 and 5.

**Table 2:** Comparison of Functional Outcomes Depending of type of Defect (mean ± SD, CI 95%).

Functional Scale	IIB (n = 22)	IIIA (n = 37)	IIIB (n = 10)	p-value (IIB vs IIIA)	p-value (IIB vs IIIB)	p-value (IIIA vs IIIB)
HHS	89.7(± 2.8)	86.9(± 3.7)	79.2(± 5.6)	0.0018*	0.0002*	0.0017*
WOMAC	24.4(± 2.06)	26.0(± 2.0)	25.3(± 1.2)	0.0049*	0.06 (ns)	0.32 (ns)
VAS	3.5(± 0.4)	3.4 ± 0.6	3.25(± 0.8)	0.54 (ns)	0.21 (ns)	0.46 (ns)

### Implant survivorship

**Fig. 6:**
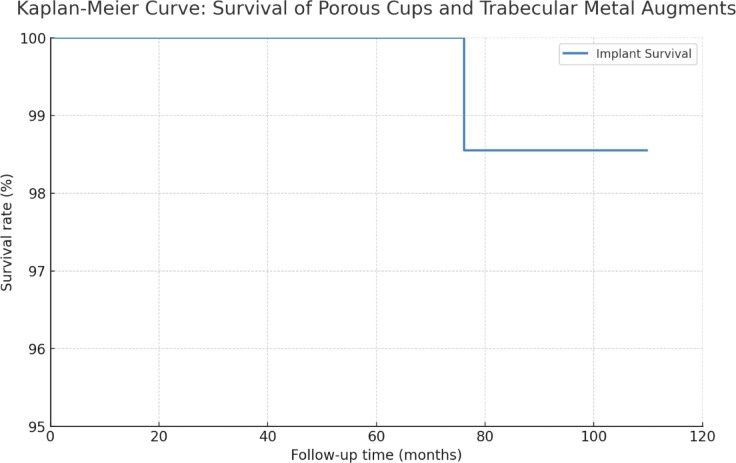
Survival analysis of porous cups and trabecular augments (Kaplan–Meier method).

Kaplan–Meier survival analysis demonstrated a 9 years (109.7 months) cumulative implant survivorship rate of 98.55% (Fig. 6). No patients were lost to follow-up throughout the observation period.

**Fig. 7:**
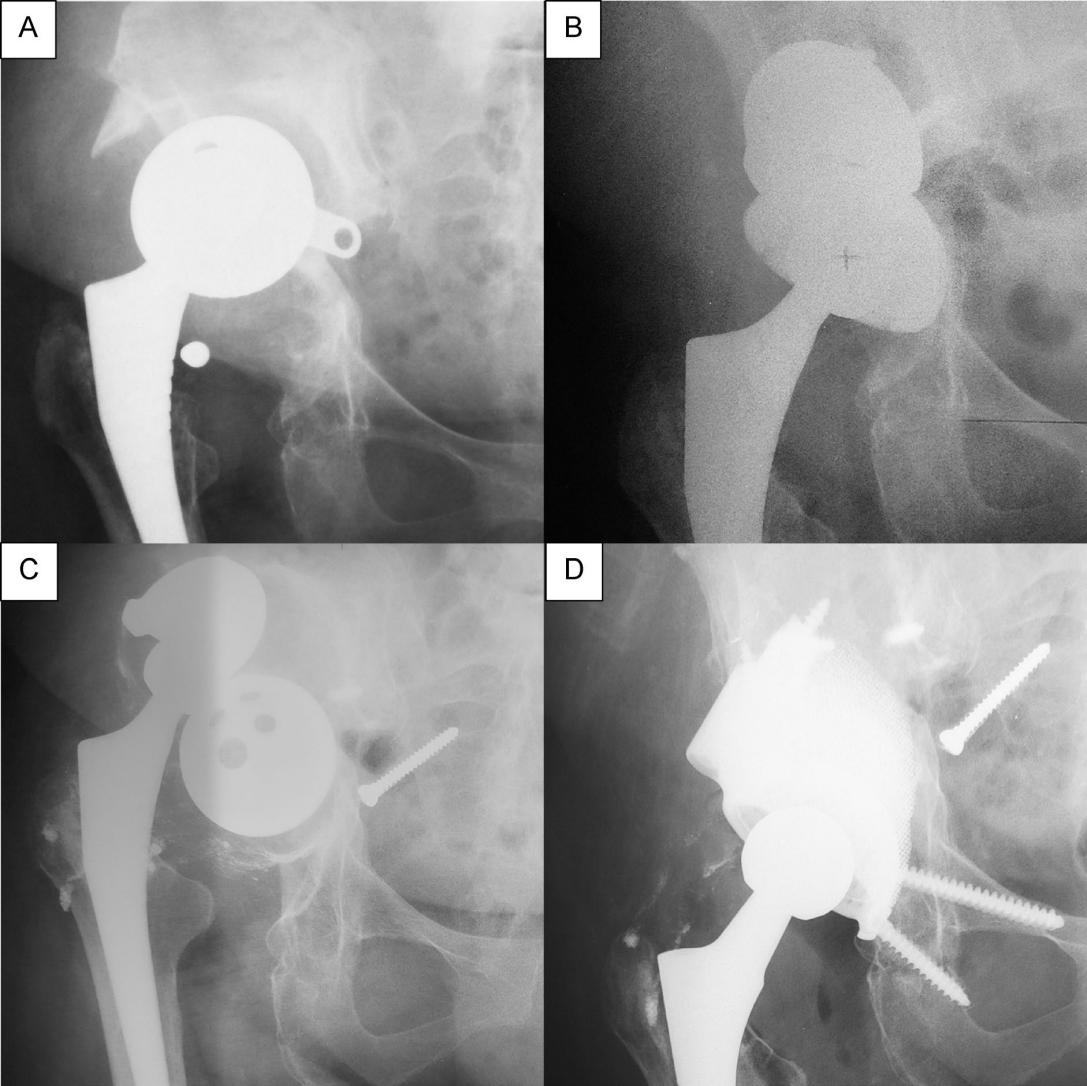
Case of aseptic loosening of the patient from the study cohort. (**A**) Preoperative radiograph demonstrating severe cranial migration of the acetabular component and a Paprosky type IIIB defect. (**B**) Immediate postoperative radiograph following reconstruction with a porous cup and trabecular metal augment. (**C**) Radiograph at 6.3-year follow-up showing signs of aseptic loosening of the construct. (**D**) Re-revision procedure performed using a custom-made 3D acetabular implant.

A single case of re-revision surgery was recorded in a patient with a Paprosky type IIIB acetabular defect at 76.1 months postoperatively due to progressive aseptic loosening of the acetabular component (Fig. 7). It is noteworthy that restoration of the anatomical center of rotation could not be fully achieved during the initial revision procedure, which may have contributed to the mechanical failure and was considered a plausible contributing factor. The subsequent re-revision was performed using a patient-specific custom-made 3D-printed implant.

**Fig. 8:**
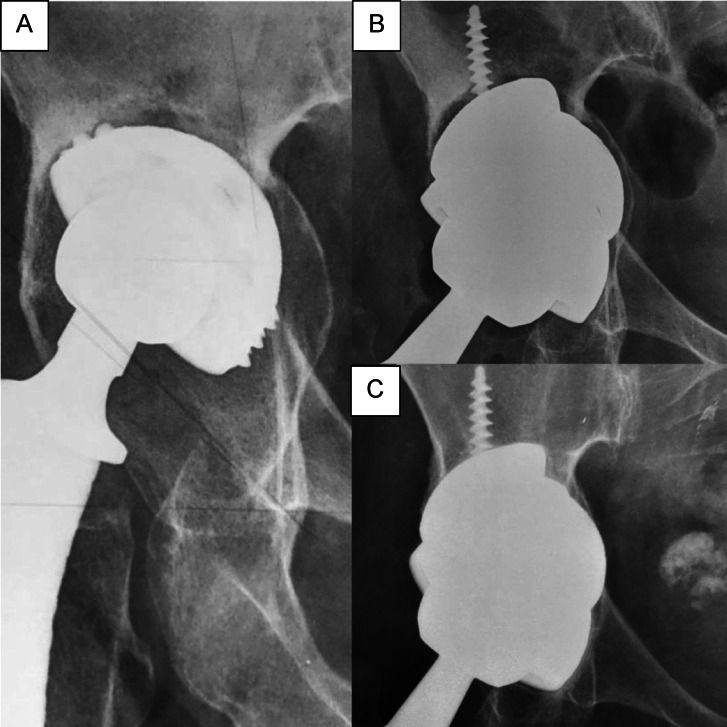
Case presentation of a patient with a type IIIB acetabular defect. (**A**) Preoperative anteroposterior pelvic radiograph demonstrating aseptic loosening and severe migration of the acetabular component. (**B**) Immediate postoperative radiograph showing reconstruction with a porous acetabular cup and trabecular metal augments in footing-position. (**C**) Radiograph at 6-year follow-up demonstrating stable component positioning with radiographic evidence of osseointegration at the bone–implant interface.

In the remaining 68 cases, there were no radiographic or clinical signs of loosening of the porous acetabular components or the trabecular augments. In the vast majority of cases, radiographic signs of stable bone integration of the trabecular metal augments were observed (Fig. 8). The mean inclination angle of the acetabular component postoperatively was 42.8° (± 2.7°) and the mean vertical position of the center of rotation relative to the inter-teardrop line was 14 mm (± 1.6 mm).

### Complications and adverse events

No complications requiring surgical revision or reoperation were observed in the study cohort. In three cases, patients developed peroneal branch sciatic nerve palsy, presenting clinically as foot drop due to impaired ankle dorsiflexion. In all cases, complete neurological recovery was achieved—within 2 months in one patient, and within 6 months in the other two.

In two cases, early postoperative Morel–Lavallée-type hematomas developed between the fascia lata and subcutaneous fat tissue; both were successfully managed with drainage and compressive dressings.

In one patient with a type IIIB acetabular defect and significant cranial migration of the acetabular component, intraoperative injury to the superior gluteal artery occurred. Hemostasis was achieved using tamponade with hemostatic sponges.

## Discussion

Our findings support the concept that porous acetabular cups combined with trabecular metal augments represent a reliable reconstructive solution in acetabular revision surgery. Kaplan–Meier analysis demonstrated a 9 years implant survivorship of 98.55%. The mean Harris Hip Score (HHS) significantly improved from a preoperative mean of 32.9 (± 9.8) to 86.7 (± 4.9).

Moreover, functional outcomes according to HHS were directly correlated with the severity of acetabular bone defects, showing statistically significant differences: patients with preoperative type IIB defects had superior outcomes compared to those with type IIIA and IIIB defects. Our cohort did not include cases with pelvic discontinuity, based on global experience, our preferred strategy in such scenarios involves the use of patient-specific custom-made 3D implants or cup-cage constructs. These results are consistent with numerous similar studies in the literature.

Outcomes from 157 acetabular revision cases reconstructed with porous cups combined with trabecular metal augments in three configurations: flying buttress (30 cases), dome (70 cases), and footing (57 cases), were reported^22^. In this study, the majority of patients presented with large acetabular defects classified as type IIIA (60.5%) and IIIB (33.8%), while defects of types IIA, IIB, and IIC were observed in only 5.7% of all cases. Intraoperatively, pelvis discontinuity was identified in 17 cases (10.8%) of type IIIA and IIIB defects. The 10 years implant survivorship was 95.5% without pelvis discontinuity, and 70.9% in cases with pelvic discontinuity. Mean postoperative HHS at 10 years was 79.2.

Another investigation of 104 acetabular reconstructions assessed dome and flying buttress augments, classifying bone defects according to the Gross system^23^. The authors used flying buttress configuration in 51% of cases and dome configuration in 49%. Among the seven cases of aseptic implant loosening, five occurred in the flying buttress group, while only two were observed in the dome group. This difference was statistically significant. The mean postoperative HHS was 77.3, and 10 years survivorship was 90.4%. No statistically significant differences in revision risk were observed depending on defect type.

In a separate cohort including patients with type IIIA (31 cases) and IIIB (10 cases) defects, the use of porous cup—augment constructs resulted in excellent outcomes^24^. Only one patient experienced two episodes of dislocation, both managed non-operatively. No radiographic evidence of bone resorption around the cup or augment was reported. Implant survivorship was 100%, and the mean HHS was 81.6.

Similarly, another series of 25 revision procedures, including 10 cases with type III defects (6 IIIA, 4 IIIB), demonstrated 100% Kaplan–Meier survivorship at 10 years, with a mean HHS of 91.05, and only one patient (4%) dissatisfied with functional outcome^25^.

Despite the demonstrated efficacy of trabecular augments and porous cups, alternative reconstructive options remain in use, such as anti-protrusion reinforcement rings (e.g., Burch–Schneider or Ganz), which continue to provide acceptable results. However, their popularity is declining, likely due to limited osteointegration potential and suboptimal long-term outcomes. According to the findings of one comparative study with a mean follow-up of 7.5 years, survivorship was 80.8% for Burch-Schneider rings and 91.2% for trabecular metal cups combined with augments or allografts, favoring the latter in cases with large bone defects^26^.

Further analyses of reinforcement rings have provided additional long-term data. In one series, Burch–Schneider rings were applied in 14 cases with IIIA and IIIB defects (including one case with pelvic discontinuity), while Ganz rings were used in 16 cases with IIA–IIC defects. At 15 years of follow-up, the overall Kaplan–Meier survivorship was 86.7%^27^.

Another investigation focusing on Burch–Schneider rings reported a 10 years survivorship of 73.2%, which was considered acceptable overall but markedly poorer in patienes with type IIIB defects. The authors concluded that Burch-Schneider anti-protrusion rings are an acceptable option for the treatment of type IIC and IIIA defects; however, for the reconstruction of type IIIB defects, they prefer to consider alternative options such as custom-made 3D implants or porous cups in combination with trabecular metal augments^28^.

The combination of Burch–Schneider rings with structural allografts has also been explored in complex reconstructions, including defects type IIIA (17 cases), IIIB (12 cases), and pelvic discontinuity (2 cases). Reported implant survivorship was 76% at 5 years and 57% at 10 years. The authors concluded that this approach is technically demanding and carries a high complication risk. Nonetheless, even in cases of implant failure, successful re-revision using porous acetabular components remains feasible due to integration of the structural graft^29^.

Collectively, these data reflect a growing trend toward favoring porous cup–augment constructs for acetabular defect reconstruction. A growing body of evidence and expert opinion among orthopedic surgeons support their efficacy in complex revision scenarios.

There are several potential limitations of this study. Although the data were collected prospectively, their retrospective analysis and lack of a control group increase the risk of bias. The current mean follow-up duration does not allow for definitive conclusions regarding the long-term durability of porous cups and trabecular augments, necessitating continued follow-up. The patient cohort size remains relatively small, limiting the generalizability of our findings. Moreover, although all surgeries were performed by three experienced surgeons within a single institution following a standardized protocol, individual variations in surgical technique, defect classification, and component positioning may influence outcomes.

Despite a robust data collection protocol and prospective patient monitoring, five patients from remote areas did not provide follow-up radiographs, although they reported no complaints and achieved excellent HHS and WOMAC scores.

All aforementioned factors may potentially impact final outcome interpretation.

## Conclusion

Porous acetabular cups in combination with trabecular metal augments represent a highly effective solution in acetabular revision surgery, providing excellent mid-term implant survivorship and favorable functional outcomes. The favorable clinical and radiological mid-terms outcomes observed in this study support the continued use of porous cups combined with trabecular augments in revision procedures. Further long-term follow-up studies are warranted to confirm the sustained success of this technique over time.

## Data Availability

The data and materials used and analyzed during this study are available from the corresponding author and from the registry of the Department of Joint Arthroplasty at the Priorov National Medical Research Center of Traumatology and Orthopedics.

## Abbreviations

3D: Three dimentional
THA: Total hip arthroplasty
HHS: Harris hip score
VAS: Visual Analog scale
WOMAC: Western Ontario and mcmaster universities arthritis index
SPSS: Statistical package for the social sciences
